# A machine learning analysis of a “normal-like” IDH-WT diffuse glioma transcriptomic subgroup associated with prolonged survival reveals novel immune and neurotransmitter-related actionable targets

**DOI:** 10.1186/s12916-020-01748-x

**Published:** 2020-10-16

**Authors:** H. D. Nguyen, A. Allaire, P. Diamandis, M. Bisaillon, M. S. Scott, M. Richer

**Affiliations:** 1 Department of Biochemistry and Functional Genomics, Université de Sherbrooke, Sherbrooke, Québec Canada; 2 Department of Laboratory Medicine and Pathobiology and Princess Margaret Cancer Center, University of Toronto, Toronto, Ontario Canada; 3 Department of Pathology, Université de Sherbrooke, Sherbrooke, Québec Canada

**Keywords:** IDH-WT, Glioma, Transcriptomic, Biomarkers, Amino acid neurotransmission, Tumor immune checkpoints, SLC32A1, MSR1, C5AR1, SYT5

## Abstract

**Background:**

Classification of primary central nervous system tumors according to the World Health Organization guidelines follows the integration of histologic interpretation with molecular information and aims at providing the most precise prognosis and optimal patient management. According to the cIMPACT-NOW update 3, diffuse isocitrate dehydrogenase-wild type (IDH-WT) gliomas should be graded as grade IV glioblastomas (GBM) if they possess one or more of the following molecular markers that predict aggressive clinical course: EGFR amplification, TERT promoter mutation, and whole-chromosome 7 gain combined with chromosome 10 loss.

**Methods:**

The Cancer Genome Atlas (TCGA) glioma expression datasets were reanalyzed in order to identify novel tumor subcategories which would be considered as GBM-equivalents with the current diagnostic algorithm. Unsupervised clustering allowed the identification of previously unrecognized transcriptomic subcategories. A supervised machine learning algorithm (*k*-nearest neighbor model) was also used to identify gene signatures specific to some of these subcategories.

**Results:**

We identified 14 IDH-WT infiltrating gliomas displaying a “normal-like” (NL) transcriptomic profile associated with a longer survival. Genes such as C5AR1 (complement receptor), SLC32A1 (vesicular gamma-aminobutyric acid transporter), MSR1 (or CD204, scavenger receptor A), and SYT5 (synaptotagmin 5) were differentially expressed and comprised in gene signatures specific to NL IDH-WT gliomas which were validated further using the Chinese Glioma Genome Atlas datasets. These gene signatures showed high discriminative power and correlation with survival.

**Conclusion:**

NL IDH-WT gliomas represent an infiltrating glioma subcategory with a superior prognosis which can only be detected using genome-wide analysis. Differential expression of genes potentially involved in immune checkpoint and amino acid signaling pathways is providing insight into mechanisms of gliomagenesis and could pave the way to novel treatment targets for infiltrating gliomas.

## Background

Infiltrating gliomas are the most frequent malignant primary neoplasms of the central nervous system (CNS) in adults [1]⁠. They are relentlessly recurring and lethal tumors despite aggressive multimodal treatment (chemotherapy and/or radiotherapy) [2, 3]⁠. Survival of patients with infiltrating gliomas is generally short, but a unique subset of rare cases (5%) survive past 5 years despite being histopathologically diagnosed as glioblastomas [1, 4]⁠⁠.

Histological analysis is now complemented with molecular information into integrated diagnoses that provide increased standardization and prognostic reliability, as recommended in the most recent edition of the World Health Organization (WHO) classification of tumors of the central nervous system [5–7]⁠. For example, separate categories have been created based on the presence of alterations such as isocitrate dehydrogenase-wild type 1/2 (IDH1/2) [8]⁠ and histone 3 mutations, which are frequently found in adult low-grade and pediatric high-grade gliomas, respectively [9, 10]⁠.

The creation of the consortium to Inform Molecular and Practical Approaches to CNS Tumor Taxonomy (cIMPACT-NOW) is an initiative that facilitates the communication of WHO classification updates to the neuropathology community [11]⁠. For IDH-WT glioma grading, the cIMPACT update 3 recommends the assessment of EGFR amplification, combined chromosomes 7p gain/10q loss, and TERT promoter mutation, which are established predictors of poor outcome, regardless of histology [12]⁠.

The transcriptome remains underutilized as a diagnostic tool for glioma despite its great potential, as it contains complementary information on transcriptional events [13]⁠ such as RNA alternative splicing. This study sought to analyze the IDH-WT glioma expression data from The Cancer Genome Atlas (TCGA) [14]⁠ glioblastoma multiforme (GBM) [15, 16]⁠ and low-grade glioma (LGG) [17]⁠ cohorts, using machine learning algorithms, as a means to identify novel expression-based signatures with potential clinical utilities which would also provide novel insight on gliomagenesis. Unsupervised clustering identified a subgroup of 14 IDH-WT infiltrating gliomas out of a total of 238 (5%) displaying what we coin a “normal-like” (NL) transcriptomic profile associated with a superior prognosis compared to other subgroups. NL IDH-WT gliomas are partially comprised in subgroups described previously in major glioma papers but were not thoroughly characterized. In our study, we aimed at better characterizing the epidemiology and molecular profile of these atypical tumors, with an emphasis on the coding transcriptome. We identified two gene signatures composed of SLC32A1/MSR1 and SYT5/C5AR1 gene combinations whose expression alone strongly correlated with this subgroup of IDH-WT gliomas.

## Methods

### Samples

Illumina HiSeq RNASeqV2 data was downloaded from the NIH GDC Data Portal [18]⁠ (https://portal.gdc.cancer.gov). Only IDH-WT tumors from the low-grade glioma (LGG-TCGA) and glioblastoma (GBM-TCGA) cohorts with available expression data were included, i.e., 144 of 617 cases and 94 of 516 cases, respectively. Five normal brain control tissues, also downloaded from TCGA database, were added. Only one sample was kept for cases with multiple replicates.

### Gene expression analysis

The DESeq2 R package [19]⁠ was used to identify the differentially expressed genes between conditions. Two differential expression analyses were performed using raw HTSeq counts: normal tissues vs cancer tissues (used for the unsupervised clustering); NL cluster vs OT cluster (used for the machine learning pipeline).

Differential gene expression was performed by calculating *p* values (false discovery rate adjusted, or FDR) with the DESeq2 R package. Log2 fold changes (FC) were also calculated, and a specific threshold was selected in order to determine cluster-specific genes: FDR adjusted *p* value less than 0.001 and log2 of fold change greater than 2 or lower than − 2.

### Unsupervised clustering analysis of RNA-seq data

RNA-seq Fragment Per Kilobase per Million reads mapped (FPKM) estimates obtained using HTseq [20]⁠ were clustered in an unsupervised manner using the R package hclust, according to transcript abundance profile similarity. The dataset was filtered to include only significant differentially expressed genes between normal and cancer samples (3903 genes with FDR adjusted *p* value less than 0.001 out of 19,107 coding genes in total). This was followed by log2 standardization (with a pseudocount of 0.01) and hierarchical clustering using Pearson’s correlation. A heatmap was subsequently generated with the ComplexHeatmap R package [21]⁠. Additional file 1: Figure S1 depicts the analysis pipeline.

### Tumor purity calculation

Estimate R package [22]⁠ was used to evaluate tumor purity for each TCGA IDH-WT glioma sample (*n* = 238). This tool infers tumor purity from the expression of stromal and immune cell markers in tumor tissues.

### Clinical data analysis

Survival data, made available from NIH GDC Data and TCGA portals, were analyzed with survival and survminer R packages [23, 24]⁠. In addition, we performed chi-squared statistical tests on other clinical data such as age at diagnosis, gender, and vital status. Relative survival curves and log-rank tests were computed for identified transcriptomic subgroups.

### Copy number variation analysis

The GISTIC2 v.2.0 software was used to identify significant chromosomal aberrations such as deletions and amplifications [25]⁠. Masked copy number variation data for the different transcriptomic clusters were analyzed individually with TCGA GISTIC2 pipeline parameters [18]⁠. Copy number variation (CNV) data associated with Y chromosomal aberrations in germinal cells were excluded from this analysis. False discovery rate (FDR) values were calculated for each chromosomal aberration.

### Histological review

Scanned slides from cases included in our cohort of NL IDH-WT gliomas were reviewed by two Canadian Board-certified diagnostic neuropathologists.

### Machine learning pipeline for gene identification signatures

Raw HTSeq counts from differentially expressed genes between normal and cancer samples (3903 genes) used for heatmap generation were further analyzed for gene expression comparison between identified transcriptomic clusters. The set was filtered to 3806 genes by only keeping genes with an expression of at least 1 count in at least 75% of tissue samples. From this filtered set, we selected genes that are differentially expressed between identified clusters associated with a FDR adjusted *p* value lower than 0.001 and log2 of fold change greater than 2 or below − 2.

Pseudocounts of 0.01 were added to the reduced FPKM expression data, which were then log2 transformed. The dataset was then randomly split into training and test sets with 80% (190/238) and 20% (48/238) of all IDH-WT glioma tumor samples, respectively. From the training set, we extracted relevant genes with strong discrimination power (Mean Decrease Gini or MDG, based on Gini index) between the different transcriptomic clusters using random decision forests, taking the first 50 genes with the best mean of IncNodePurity values for 100 random forests [26, 27]⁠. Relevant genes were subsequently subjected to a *k*-nearest neighbor (KNN) algorithm [28]⁠ (Scikit Learn Python library [29]⁠), using different gene combinations (combination of 1, 2, or 3 genes). Stratified cross-validation in 10 folds was also performed to determine gene combinations that allow classification of tumors associated with the different transcriptomic clusters with a minimized error rate and a maximized area under the receiver operating characteristic (ROC) curve, specificity, and sensitivity. We tested gene signatures using the test set, and ROC curves were generated to compute performance metrics using the Python package sklearn.metrics.roc_curve [29]⁠. This step is described in Additional file 2: Figure S2.

### Validation of gene signatures

The validation was performed using two independent glioma expression datasets retrieved from the Chinese Glioma Genome Atlas (CGGA [30]⁠; http://www.cgga.org.cn/ [31–33]⁠). We extracted IDH-WT glioma samples associated with survival and expression data and used the same method of standardization by log2 (adding a pseudocount of 0.01) for these expression datasets, and classification was subsequently achieved using a KNN trained with TCGA data and using identified gene signatures (see Additional file 3: Figure S3).

### Estimation of the immune cell composition

The Timer2 web server (http://timer.cistrome.org/ [34, 35]) was used to infer the relative representation of the different hematopoietic cells present within each glioma sample. This web-based tool uses the immunedeconv R package [36] which regroups six immune estimation algorithms: TIMER [37]⁠, Cibersort [38]⁠, Epic [39]⁠, quanTIseq [40]⁠, xCell [41]⁠, and MPC-counter [42]⁠. In this project, immune cell estimation values generated by Cibersort, Epic, and quanTIseq allowed the comparison between each immune cell type within the same sample. Statistical significance was determined using the Mann-Whitney *U* test with *p* < 0.05 as a threshold for at least two of the three tools.

### Statistical analysis

The enrichment analysis was performed using a chi-square test or a Fisher exact test. The Cramer test was used to measure the association between the cluster type and histological variables (tumor type and grade). Statistical differences between expression and histological variables were evaluated using the non-parametric Mann-Whitney *U* test. Log-rank tests were used for comparison of survival between tumor types. Univariate and multivariate Cox regressions were performed to validate gene signature independence using “survminer” R package [24]⁠.

## Results

### Global profiling of IDH-WT glioma gene expression and identification of a normal-like glioma cluster

**Fig. 1:**
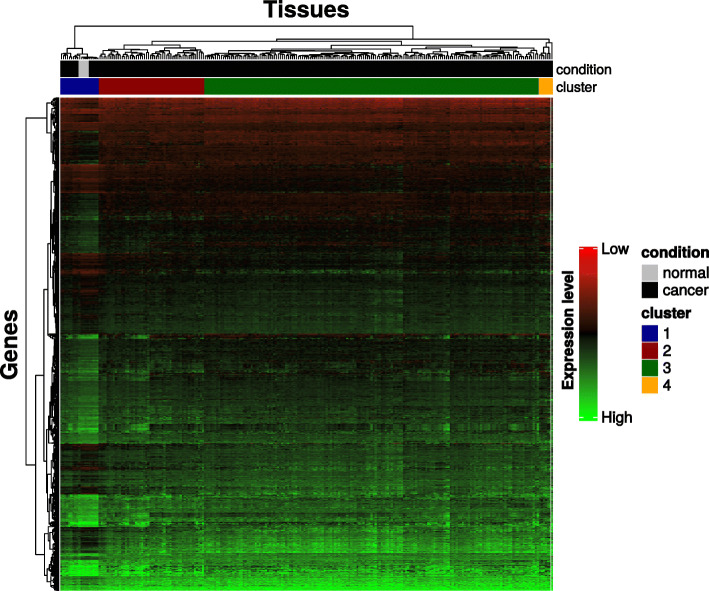
Unsupervised clustering of TCGA expression data associated with 243 samples. The 243 samples are composed of 238 IDH-WT gliomas and 5 healthy samples. We used 3903 genes differentially expressed between normal and tumor samples in this analysis, and the clustering ordering was performed using a Pearson correlation

To investigate the extent of variability in gene expression of IDH-WT gliomas, we performed unsupervised clustering of merged TCGA low-grade glioma (LGG-TCGA) and glioblastoma (GBM-TCGA) gene expression datasets comprised of 3903 differentially expressed genes for 238 IDH-WT gliomas and 5 normal brain control tissues, as depicted in Fig. 1. The heatmap generated on this filtered dataset yielded four distinct clusters with specific gene expression patterns (blue, *n* = 14; red, *n* = 52; green *n* = 165; orange *n* = 7). All five normal tissues classifed with blue cluster tumors were thus renamed “normal-like” (NL), while the red, green, and orange clusters were pooled into one group identified as “other tumors” (OT).

In previous studies, TCGA and Ceccarelli et al. analyzed and identified different clusters from TCGA data [17, 43]⁠. These tumor categories were derived using the whole TCGA dataset, composed of heterogeneous data such as RNA-seq, methylation, miRNA, and copy number data for IDH-WT and IDH-mutant gliomas. For TCGA study, molecular subcategories were the following: R1–R4 (RNAseqClusters), M1–M5 (MethylationClusters), mi1–mi4 (miRNAClusters), and C1–C3 (CNClusters). For the Ceccarelli study, these included LGr1–4 (Pan-Glioma RNA expression Clusters) and LGm1–6 (Pan-Glioma DNA methylation Clusters).

**Fig. 2:**
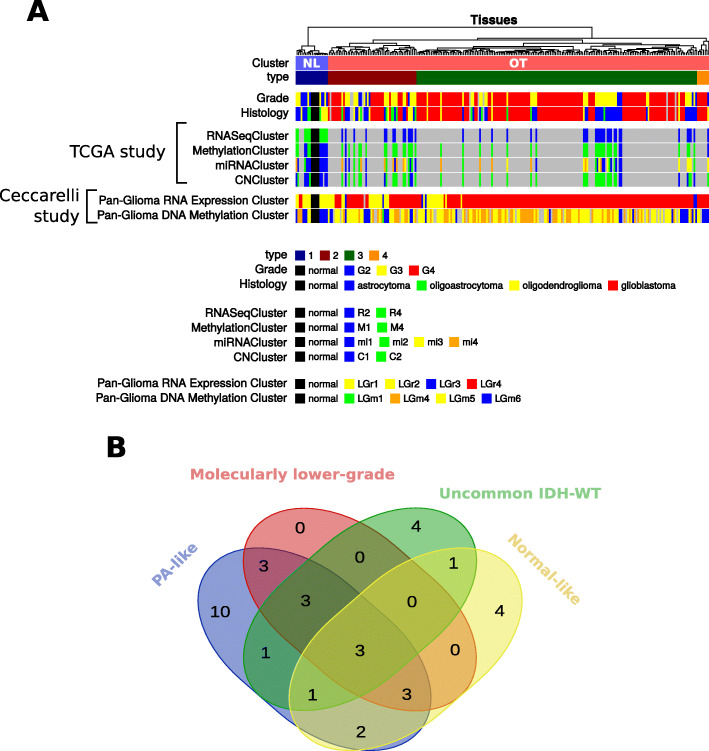
Comparison analysis with TCGA, Ceccarelli, and Aibaidula studies. **a** Comparison of the identified clusters with tumor subgroups identified in TCGA and Ceccarelli studies. The color code for the different clusters is provided at the bottom of the figure. **b** Comparison of the IDH-WT NL gliomas with previously published molecular glioma subgroups associated with longer survival only shows partial overlap (in green, uncommon IDH-WT gliomas from TCGA, 2015; in blue, pilocytic astrocytoma-like subgroup from Ceccarelli et al. 2016; in pink, molecularly low-grade from Aibaidula et al., 2017)

After identifying four main clusters with the unsupervised clustering of the IDH-WT glioma expression data (Fig. 1), we investigated whether these clusters corresponded to clusters identified in previous studies. The comparison between our clusters defined on RNA-seq data and clusters previously defined in TCGA and Ceccarelli studies is described in Fig. 2a. As compared to the partial data available in TCGA study, the majority of gliomas associated with the NL cluster were classified as R4 (11/11), M1 (7/11), mi1 (9/11), and C1 (8/10) whereas the OT cluster was heterogeneously composed of R2 (42/42), M4 (42/46), mi2–4 (14/47, 10/47, 11/47, and 12/47, respectively), and C2 (37/47) tumors. For the Ceccarelli study, the NL cluster was essentially enriched in LGr2 (12/14) and LGm6-GBM (9/14) classes whereas the LGr4 (187/223) and LGm4–6 (62/193, 100/193, and 30/193, respectively) classes were distributed uniformly in the OT cluster. These results and the associated Fisher’s exact test *p* values are presented in Table 1. Altogether, these data are in keeping with the existence of a distinct cluster of tumors showing normal-like transcriptomic profiles which are different from other IDH-WT gliomas. Furthermore, it confirms the molecular heterogeneity in these usually aggressive tumors.

**Table 1:** Distribution of TCGA and Ceccarelli study clusters associated with normal-like (NL) and other-type (OT) IDH-WT gliomas (for each category, only samples for which data are available were considered)

	NL	OT	***p*** value (Fisher’s exact test)
**RNASeqCluster (*n*** **= 53 with available data)**	***N*** **= 11**	***N*** **= 42**	
R2	0 (0.0%)	42 (100%)	1.31e−11
R4	11 (100%)	0 (0.0%)	
**MethylationCluster (*n*** **= 57 with available data)**	***N*** **= 11**	***N*** **= 46**	
M1	7 (63.6%)	4 (8.7%)	3.06e−04
M4	4 (36.4%)	42 (91.3%)	
**miRNACluster (*n*** **= 58 with available data)**	***N*** **= 11**	***N*** **= 47**	
mi1	9 (81.8%)	14 (29.8%)	1.95e−02
mi2	1 (9.1%)	10 (21.3%)	
mi3	0 (0.0%)	11 (23.4%)	
mi4	1 (9.1%)	12 (25.5%)	
**CNCluster (n = 57 with available data)**	***N*** **= 10**	***N*** **= 47**	
C1	8 (80.0%)	10 (21.3%)	7.96e−04
C2	2 (20.0%)	37 (78.7%)	
**Pan_Glioma_RNA_Expression_Cluster (*n*** **= 237 with available data)**	***N*** **= 14**	***N*** **= 223**	
LGr1	0 (0.0%)	26 (11.7%)	5.00e−04
LGr2	12 (85.7%)	4 (1.8%)	
LGr3	0 (0.0%)	6 (2.7%)	
LGr4	2 (14.3%)	187 (83.9%)	
**Pan_Glioma_DNA_Methylation_Cluster (*n*** **= 207 with available data)**	***N*** **= 14**	***N*** **= 193**	
LGm1	0 (0.0%)	1 (0.5%)	1.50e−03
LGm4	3 (21.4%)	62 (32.1%)	
LGm5	2 (14.3%)	100 (51.8%)	
LGm6	9 (64.3%)	30 (15.5%)	

In addition, we compared our clusters to previous classifications identifying gliomas with a better prognosis. Two previous studies from Aibaidula and colleagues [44]⁠ had identified a minority of IDH-WT gliomas associated with longer survival using the same TCGA dataset [18]⁠ (LGG and GBM projects). These atypical gliomas were respectively labeled as “uncommon IDH-WT,” “PA-like,” and “molecularly low-grade” in TCGA, Ceccarelli, and Aibaidula studies. Comparisons with these studies (Fig. 2b) showed that the NL cluster identified in our analyses (*n* = 14) was significantly enriched in uncommon IDH-WT (5/14, *p* value = 1.78e−06), PA-like (9/14, *p* value = 7.47e−10), and molecularly low-grade (6/14, *p* value = 1.58e−09) tumors. However, the overlap with these previously mentioned categories was partial, with 4 cases belonging only to the NL subgroup.

This comparison analysis showed that the NL cluster possesses a specific transcript abundance profile when compared to the OT cluster which displayed more heterogeneous profiles (Fig. 2a). For the rest of the study, we thus decided to characterize the differences between the NL (*n* = 14) and OT tumors (*n* = 224).

### NL tumor purity

We verified the possibility that the normal-like profile was related to low tumor cell density by evaluating purity with the R packages “ESTIMATE,” which is based on the estimation of stromal and immune cell markers [22]⁠. Indeed, the normal-like profile could possibly be explained by tumor cell dispersion in brain parenchymal non-neoplastic cells which would, in turn, skew the expression pattern in these tumors and explain their normal-like profile.

**Fig. 3:**
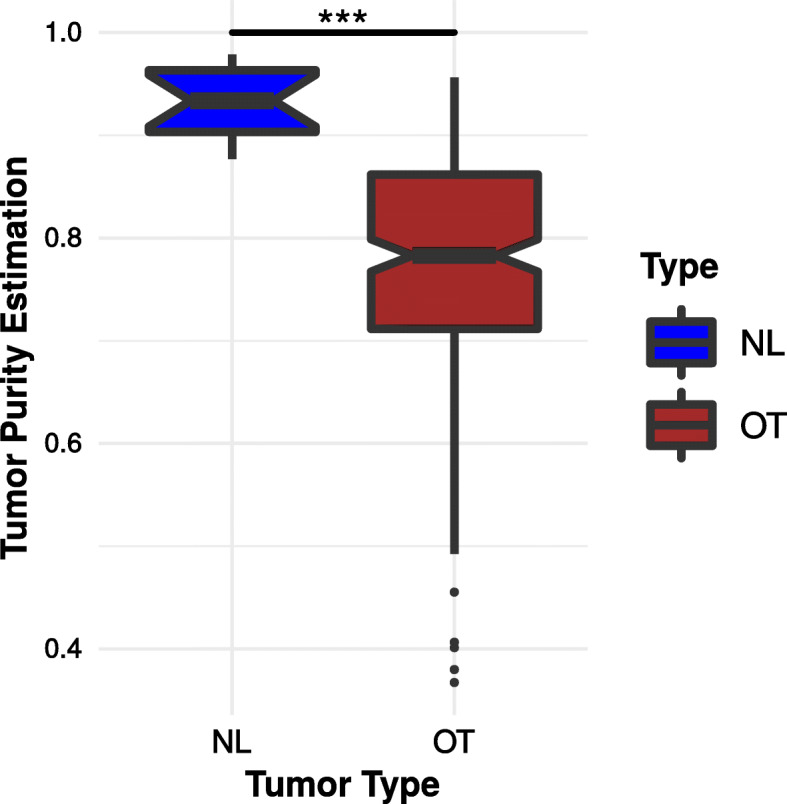
Comparison of tumor purity estimation for NL and OT IDH-WT tumors. The tumor purity was estimated with the R package ESTIMATE. Statistical analysis was performed with the Wilcoxon signed-rank test

The NL IDH-WT tumors showed significantly increased purity estimation scores when compared to OT tumors (mean 0.93 vs 0.77; median 0.93 vs 0.78, *p* = 4.26e−08; Fig. 3 and Additional file 4: Table S1), thus demonstrating that the transcriptomic profile of this subgroup is minimally affected by non-neoplastic cells.

### NL tumors are associated with a longer overall survival

**Fig. 4:**
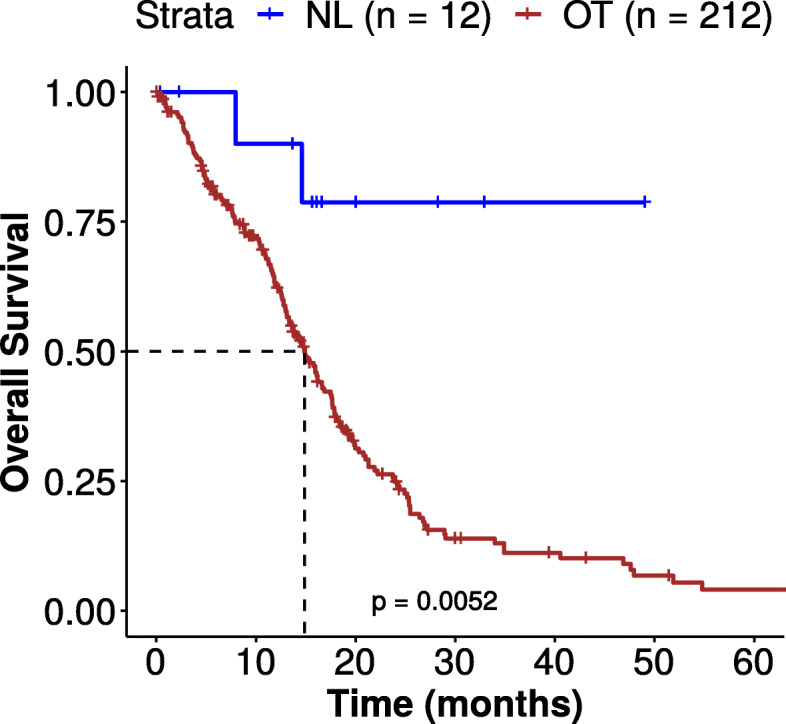
Kaplan-Meier survival curves for NL vs OT tumors identified in TCGA transcriptomic dataset. NL IDH-WT tumors are associated with a better survival profile (*p* value < 0.05 (*); *p* value = 0.0052). Statistical analysis was performed with the log-rank test

The discovery of a NL IDH-WT subgroup associated with a nearly normal transcriptomic profile may suggest a potentially better clinical outcome. The survival analysis showed that patients ascribed to the NL cluster had a longer survival than OT patients (*p* = 0.052 with a log-rank test, Fig. 4). We observed a median survival of 14.9 months for the OT group whereas the survival rate of the NL group did not drop to 50% survival.

Statistical differences were investigated between NL and OT tumors for the following epidemiological variables (Table 2): age at diagnosis, gender, vital status, and Karnofsky performance status (KPS).

**Table 2:** Epidemiological characteristics associated with the NL and OT IDH-WT glioma clusters

IDH-WT gliomas (***n*** = 238)	NL (***n*** = 14)	OT (***n*** = 224)
**Age at diagnosis (years)**		
Mean	43	58.9
Standard deviation	20.8	12.8
Min	21	23
Median	40.5	60
Max	87	89
Unknown	0	9
**Gender**		
Female	6 (42.9%)	84 (37.5%)
Male	8 (57.1%)	131 (58.5%)
Unknown	0 (0%)	9 (4%)
**KPS**		
100	0 (0%)	17 (7.6%)
90	2 (14.3%)	20 (8.9%)
80–70	3 (21.4%)	84 (37.5%)
< 70	0 (0%)	36 (16.1%)
Unknown	9 (64.3%)	67 (29.9%)

OT patients were significantly older than NL patients (*p* = 2.0e−02 by Wilcoxon-Mann-Whitney test) whereas the gender was not significant between these groups. In addition, we found that the NL group had significantly more patients alive than the OT group (71.4% vs 24.1%, *p* = 6.66e−05 with chi-square test). The KPS index was not significantly different across the two groups.

### Histological characteristics associated with the NL cluster gliomas

**Fig. 5:**
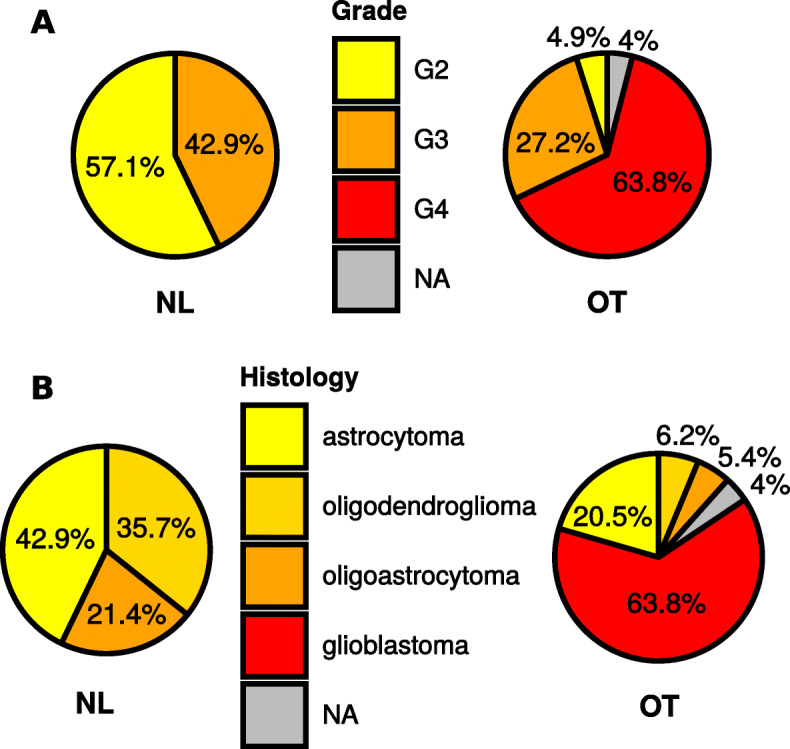
Histological distribution in NL vs OT IDH-WT gliomas. Distribution of tumor grade (**a**) and tumor histology (**b**) in NL gliomas (*n* = 14) vs other IDH-WT gliomas (*n* = 224). Cramer’s *V* with the chi-square test was used to measure the association between tumor type (NL/OT) and tumor grade/histology. Morphological oligoastrocytomas are included in TCGA dataset, as per the 2007 WHO classification of CNS tumors. Those included in this cohort would be classified as astrocytomas in the 2016 classification scheme, based on the absence of IDH1/2 and 1p/19q alterations

Because the NL gliomas display strong differences in their expression profiles and survival rates as compared to OT gliomas, we decided to consider the histological diagnoses associated with the cases in our cohort. NL cluster tumors were exclusively comprised of grade II (57.1%) and grade III tumors (42.9%). The OT IDH-WT glioma category was composed of grade III (27.2%) and grade IV gliomas (63.8%). The Cramer test, based on chi-square statistic and which measures the degree of association between two nominal variables, showed a strong correlation between the group type and the grade (*p* = 2.90e−12, Cramer’s *V* = 0.48, Fig. 5a).

Similarly, tumor histology was significantly different between NL and OT subgroups (*p* = 1.07e−06, Cramer’s *V* = 0.37, Fig. 5b): while NL tumors were a mixture of astrocytomas (42.9%), oligoastrocytomas (21.4%), and oligodendrogliomas (35.7%), OT tumors were, for the majority, histologically classified as glioblastomas (63.8%).

Scanned slides available on TCGA platform were reviewed for all NL tumor cases and did not reveal any specific features which would support that they represent a specific tumor category on histological grounds.

### Prevalence of common glioblastoma genetic alterations in NL gliomas

Next, we sought to analyze the mutational and alteration burden of this IDH-WT glioma subcategory associated with a longer survival and enriched in low-grade tumors. Mutation counts and genetic alterations generated in TCGA and Ceccarelli study [43]⁠ were reanalyzed for NL and OT tumors. We used GISTIC2 to analyze the copy number variation data for the identification of genomic alterations (EGFR, FGFR, MYB, MYBL, CDKN2A/B).

**Fig. 6:**
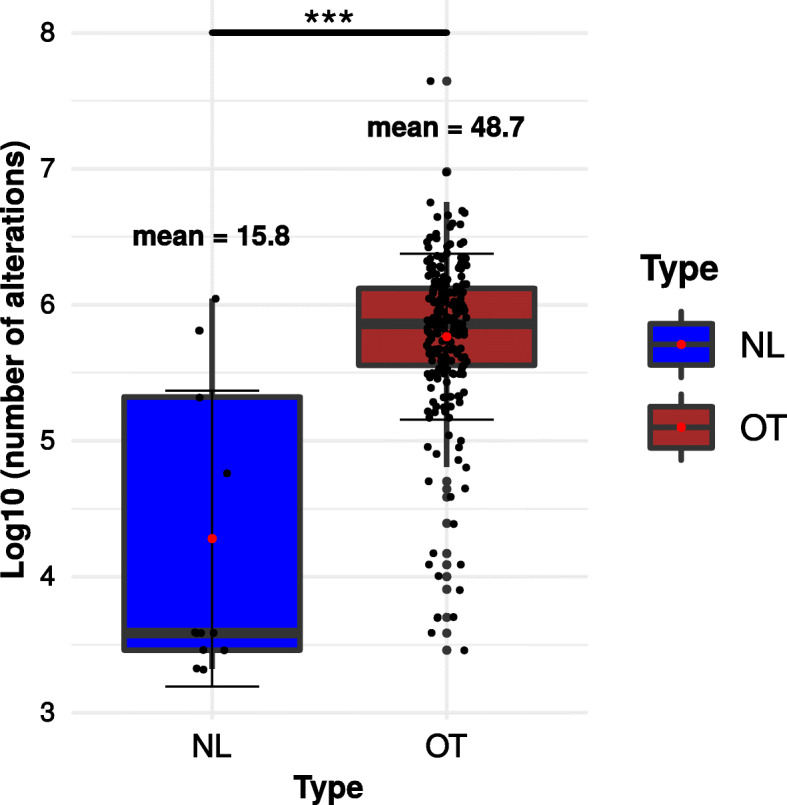
Mutation and genomic alteration profile in NL vs OT tumors. Mutation burden associated with NL vs OT tumors. The log_10_ of the number of single nucleotide variations (SNVs) obtained from the Ceccarelli study was counted for each type. Mann-Whitney *U* tests were used for the statistical analysis

A lower mutational burden was detected in NL tumors when compared to the OT tumors (*p* = 1.36e−05 with a Wilcoxon-Mann-Whitney test, Fig. 6).

The prevalence of 13 alterations typically found in gliomas is presented in Table 3. NL tumors show a lower prevalence for EGFR amplification (42.9% vs 88.4%, *p* = 8.2e−05), chr 7 gain/chr 10 loss (14.3% vs 67%, *p* = 2.2e−04), TERT promoter mutation (7.1% vs 27.7%, 68.3% with an unknown status, *p* = 4.2e−07), and CDKN2A (35.7% vs 74.1%, *p* = 2.1e−03) and CDKN2B (35.7% vs 73.2%, *p* = 2.5e−03) deletions. Six out of 14 NL gliomas would fulfill the most recent cIMPACT-NOW criteria for diffuse astrocytic glioma, IDH-WT, with molecular features of glioblastoma (grade IV). Low mutational rates for ATRX and FGFR1 were noted in both NL and OT tumors (0% and 3.6% for ATRX status, respectively), and these alterations were present with the same frequency in both groups. There was no significant difference in the MGMT methylation status between NL and OT gliomas (*p* = 0.58). A single NL case showed a BRAF V600E mutation. No BRAF-KIAA1649 fusions were present in either of the subgroups. Alterations of the following genes were only present in OT tumors: MYB (66.1% with WT status) and MYBL1 (78.6% with WT status).

**Table 3:** Distribution of genomic alterations associated with normal-like (NL) and other-type (OT) tumors

IDH-WT gliomas (***n*** = 238)	NL (***n*** = 14)	OT (***n*** = 224)	***p*** value (Fisher’s test)
**TERT promoter status**			
Mutant	1 (7.1%)	62 (27.7%)	
WT	10 (71.4%)	9 (4%)	**4.2e−07**
Unknown	3 (21.4%)	153 (68.3%)	
**Chr 7 gain/chr 10 loss**			
Gain chr 7 and loss chr 10	2 (14.3%)	150 (67%)	
No combined CNA	11 (78.6%)	70 (31.2%)	**0.00022**
Unknown	1 (7.1%)	4 (1.8%)	
**BRAF V600E status**			
Mutant	1 (7.1%)	2 (0.9%)	
WT	13 (92.9%)	218 (97.3%)	**0.17**
Unknown	0 (0%)	4 (1.8%)	
**BRAF-KIAA1549 fusion**			
Fusion	0 (0%)	1 (0.4%)	
WT	14 (100%)	217 (96.9%)	**1**
Unknown	0 (0%)	6 (2.7%)	
**ATRX status**			
Mutant	0 (0%)	8 (3.6%)	
WT	14 (100%)	212 (94.6%)	**1**
Unknown	0 (0%)	4 (1.8%)	
**EGFR**			
EGFR amplification	6 (42.9%)	198 (88.4%)	**0.000082**
EGFR deletion	0 (0%)	1 (0.4%)	
WT	8 (57.1%)	24 (10.7%)	
Unknown	0 (0%)	1 (0.4%)	
**FGFR1**			
FGFR1 amplification	0 (0%)	20 (8.9%)	**0.61**
FGFR1 deletion	1 (7.1%)	29 (12.9%)	
WT	13 (92.9%)	174 (77.7%)	
Unknown	0 (0%)	1 (0.4%)	
**CDKN2A**			
CDKN2A amplification	0 (0%)	6 (2.7%)	
CDKN2A deletion	5 (35.7%)	166 (74.1%)	**0.0021**
WT	9 (64.3%)	51 (22.8%)	
Unknown	0 (0%)	1 (0.4%)	
**CDKN2B**			
CDKN2B amplification	0 (0%)	7 (3.1%)	
CDKN2B deletion	5 (35.7%)	164 (73.2%)	**0.0025**
WT	9 (64.3%)	52 (23.2%)	
Unknown	0 (0%)	1 (0.4%)	
**MYB**			
MYB amplification	0 (0%)	6 (2.7%)	**0.12**
MYB deletion	1 (7.1%)	69 (30.8%)	
WT	13 (92.9%)	148 (66.1%)	
Unknown	0 (0%)	1 (0.4%)	
**MYBL1**			
MYBL1 amplification	0 (0%)	22 (9.8%)	**0.68**
MYBL1 deletion	1 (7.1%)	25 (11.2%)	
WT	13 (92.9%)	176 (78.6%)	
Unknown	0 (0%)	1 (0.4%)	
**MGMT_promoter_status**			
Methylated	4 (28.6%)	72 (32.1%)	
Unmethylated	10 (71.4%)	121 (54.0%)	**0.58**
Unknown	0 (0.0%)	31 (13.8%)	

### In silico identification of gene signatures for NL IDH-WT gliomas

The strong differences displayed by the NL glioma subgroup suggest that these patients would benefit from less aggressive treatment. In order to identify gliomas based on RNA-seq expression profiles, we used the *k*-nearest neighbor model to identify genes that can classify with a maximum accuracy unknown IDH-WT gliomas into the NL and OT groups. The better the classification performance of the gene signature, the better will be the separation between NL and OT samples based on the gene signature expression. Using the expression training set, we tested each combination of *n* genes (*n* = 1, *n* = 2, *n* = 3) and we selected signatures with a minimum number of genes allowing the discrimination with the best performance. Then, we selected the best signatures and tested them on the independent expression data (testing set).

We identified two 2-gene signatures, amongst 4950 tested 2-gene signatures in total, allowing the classification of the NL and OT glioma subgroups with the best performance. These signatures are composed of the SLC32A1 and MSR1 genes and the C5AR1 and SYT5 genes, respectively, and were associated with the best classification performance on the training set samples. They were validated on TCGA independent test set, in which the best classification was attained. In contrast, we obtained a lower performance of the test set classification when only one of these genes was used in the KNN model. Classification performances are shown in Additional file 5: Figure S4.

**Fig. 7:**
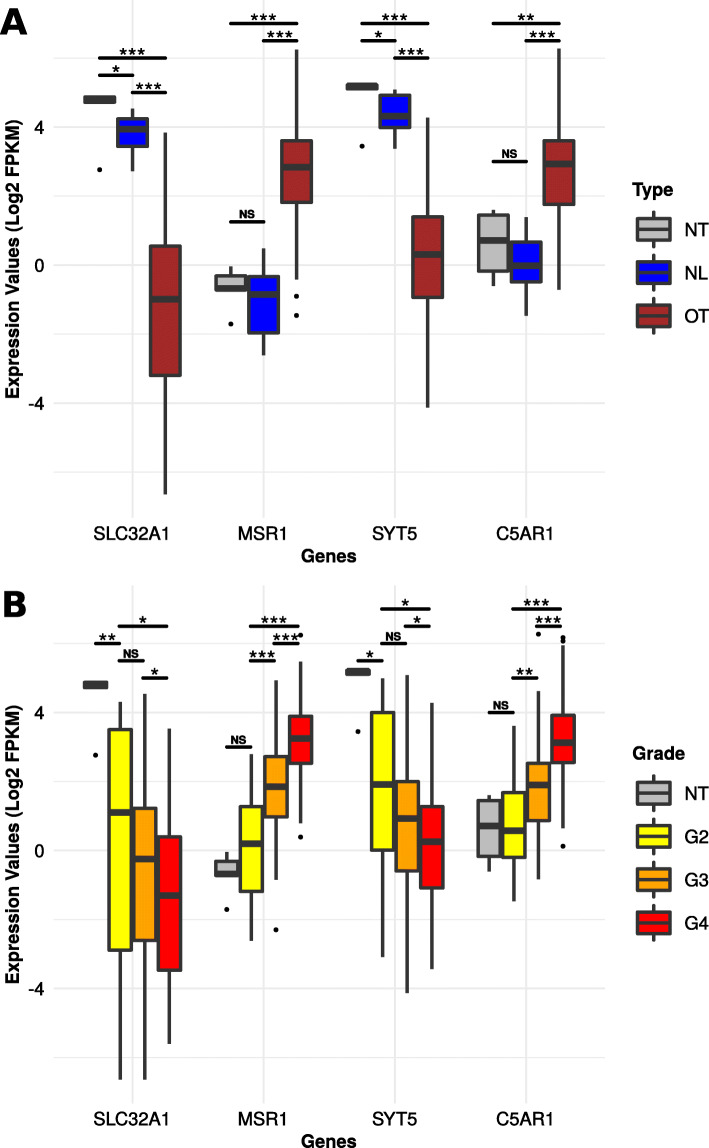
Expression levels of the C5AR1, SLC32A1, MSR1, and SYT5 genes. The boxplots show the C5AR1, SLC32A1, MSR1, and SYT5 gene expression level related to the tumor type (**a**) or the tumor grade (**b**). The NT type (in gray) corresponds to normal tissues. Statistical analysis was calculated with Mann-Whitney *U* tests (*p value < 0.05; **p value < 0.01; ***p value < 0.001)

Further characterization of these gene signatures indicated that the C5AR1 and MSR1 genes were significantly overexpressed in the OT cluster (Fig. 7a) and, in general, in higher-grade gliomas (Fig. 7b). In contrast, the SLC32A1 and SYT5 genes were underexpressed in the OT cluster and in higher-grade gliomas.

### Validation of gene signatures with the Chinese Glioma Genome Atlas

To further validate our results, we trained a KNN model with TCGA data and the two selected gene signatures independently. We then used the model to identify new NL IDH-WT gliomas from the Chinese Glioma Genome Atlas (CGGA, composed of 2 datasets *n* = 286 and *n* = 149 IDH-WT gliomas, respectively, Table 4). We selected the NL IDH-WT gliomas identified with both gene signatures.

**Table 4:** Description of external glioma databases (CGGA1 and CGGA2)

Dataset	CGGA1	CGGA2
Gliomas (with RNA-seq data)	693	325
IDH-WT gliomas (with RNA-seq data)	286	149
IDH-WT gliomas (with RNA-seq data and survival data)	277	144

**Fig. 8:**
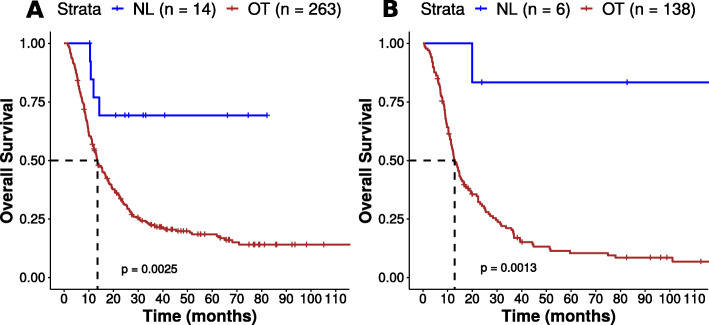
Kaplan-Meier survival curves for NL vs OT tumors identified in independent datasets. Kaplan-Meier survival curves for NL vs OT tumors identified in the first (**a**) and the second (**b**) CGGA datasets using the SLC32A1/MSR1 and C5AR1/SYT5 gene signatures. Statistical analysis was performed using a log-rank test

Using the first CGGA dataset, 14 samples were classified as NL gliomas with both SLC32A1/MSR1 and C5AR1/SYT5 gene signatures vs 263 OT samples. These 14 NL patients had a significantly longer survival than the 263 OT patients (*p* = 0.0025; median survival > 80 months vs 13.4 months; Fig. 8a). In the second CGGA dataset (*n* = 149), 6 NL gliomas were identified vs 138 OT gliomas. The survival analysis also showed a longer survival for NL patients in this dataset (*p* < 0.0013; median survival > 110 months vs 12.7 months; Fig. 8b).

### Validation of gene signatures using Cox regression analysis

To confirm the prognostic prediction power of the gene signatures, we performed univariate and multivariate Cox regression analyses. Variables with significant enrichment were added in the model: age, grade, EGFR amplification, chr 7 gain/chr 10 loss, and CDKN2A and CDKN2B deletions. One of the assumptions of the Cox regression model is that continuous covariates have to be in a linear form, as verified by plotting the Martingale residuals against the continuous covariate [45]⁠. SLC32A1 and SYT5 genes were not associated with a linear form (see Additional file 6: Figure S5), and signatures were consequently transformed into three categorical categories: high, medium, and low expression.

The univariate Cox regression showed that a medium expression of the SLC32A1 gene associated with a low expression of the C5AR1 gene was significantly associated with better prognosis (*p* value = 1.55e−02, Table 5), and the multivariate regression validated that this gene signature can be used as an independent prognostic predictor (*p* = 4.74e−02). Similar results were obtained with SYT5 gene medium expression associated with low expression of the MSR1 gene (*p* = 1.41e−03 and *p* = 3.42e−03 for the univariate and multivariate Cox regression analyses, respectively; Table 6).

**Table 5:** Univariate and multivariate Cox regression model associated with the SLC32A1/MSR1 gene signature

Characteristics	Univariate Cox regression			Multivariate Cox regression		
	Beta	HR^**a**^ (95% CI^**b**^ for HR)	***p*** value	Beta	HR^**a**^ (95% CI^**b**^ for HR)	***p*** value
**Age**	0.034	1.035 (1.021–1.048)	6.75e−07	0.02	1.020 (1.004–1.037)	1.42e−02
**Gender**						
Female	Reference					
Male	0.325	1.384 (0.991–1.932)	5.65e−02	0.073	1.076 (0.756–1.531)	6.83e−01
**Grade**						
G2	Reference					
G3	0.883	2.419 (0.850–6.881)	9.77e−02	0.547	1.728 (0.576–5.187)	3.29e−01
G4	1.75	5.752 (2.113–15.660)	6.18e−04	1.186	3.273 (1.045–10.250)	4.18e−02
**EGFR**						
Amplification	Reference					
WT	− 0.888	0.412 (0.223–0.761)	4.64e−03	− 0.337	0.714 (0.328–1.555)	3.96e−01
**CDKN2A**						
CDKN2A deletion	Reference					
CDKN2A WT	− 0.702	0.496 (0.332–0.741)	6.13e−04	− 1.691	0.184 (0.023–1.472)	1.11e−01
**CDKN2B**						
CDKN2B deletion	Reference					
CDKN2B WT	− 0.653	0.520 (0.350–0.774)	1.25e−03	1.276	3.584 (0.465–27.617)	2.21e−01
**`Chr 7 gain/chr 10 loss**						
`Chr 7 gain/chr 10 loss combined CNA	Reference					
`Chr 7 gain/chr 10 loss no combined CNA	− 0.381	0.683 (0.482–0.968)	3.20e−02	0.022	1.023 (0.682–1.533)	9.14e−01
**SLC32A1/MSR1 signature**						
SLC32A1_low–MSR1_low	− 0.172	0.842 (0.385–1.842)	6.67e−01	0.304	1.355 (0.568–3.233)	4.94e−01
SLC32A1_medium–MSR1_low	− 0.91	0.403 (0.193–0.841)	1.55e−02	− 0.801	0.449 (0.203–0.991)	4.74e−02
SLC32A1_high–MSR1_low	− 0.766	0.465 (0.283–0.762)	2.41e−03	0	1.000 (0.554–1.808)	9.99e−01
SLC32A1_low–MSR1_high	0.136	1.145 (0.728–1.800)	5.57e−01	− 0.109	0.897 (0.552–1.456)	6.60e−01
SLC32A1_medium–MSR1_high	− 0.149	0.862 (0.566–1.313)	4.89e−01	− 0.084	0.920 (0.584–1.448)	7.18e−01

^a^Hazard ratio

^b^Confidence interval

**Table 6:** Univariate and multivariate Cox regression model associated with the SYT5/C5AR1 gene signature

Characteristics	Univariate Cox regression			Multivariate Cox regression		
	Beta	HR^**a**^ (95% CI^**b**^ for HR)	***p*** value	Beta	HR^**a**^ (95% CI^**b**^ for HR)	***p*** value
**Age**	0.034	1.035 (1.021–1.048)	6.75e−07	0.022	1.023 (1.006–1.039)	5.90e−03
**Gender**						
Female	Reference					
Male	0.325	1.384 (0.991–1.932)	5.65e−02	0.165	1.179 (0.823–1.689)	3.69e−01
**Grade**						
G2	Reference					
G3	0.883	2.419 (0.850–6.881)	9.77e−02	0.557	1.745 (0.587–5.189)	3.16e−01
G4	1.75	5.752 (2.113–15.660)	6.18e−04	0.901	2.461 (0.820–7.389)	1.08e−01
**EGFR**						
Amplification	Reference					
WT	− 0.888	0.412 (0.223–0.761)	4.64e−03	− 0.391	0.677 (0.314–1.459)	3.19e−01
**CDKN2A**						
CDKN2A deletion	Reference					
CDKN2A WT	− 0.702	0.496 (0.332–0.741)	6.13e−04	− 1.903	0.149 (0.018–1.212)	7.51e−02
**CDKN2B**						
CDKN2B deletion	Reference					
CDKN2B WT	− 0.653	0.520 (0.350–0.774)	1.25e−03	1.399	4.053 (0.511–32.157)	1.85e−01
**`Chr 7 gain/chr 10 loss**						
`Chr 7 gain/chr 10 loss combined CNA	Reference					
`Chr 7 gain/chr 10 loss no combined CNA	− 0.381	0.683 (0.482–0.968)	3.20e−02	0.006	1.006 (0.673–1.502)	9.78e−01
**SYT5/C5AR1 signature**						
SYT5_low–C5AR1_low	− 0.646	0.524 (0.250–1.100)	8.77e−02	− 0.536	0.585 (0.271–1.266)	1.73e−01
SYT5_medium–C5AR1_low	− 0.858	0.424 (0.250–0.718)	1.41e−03	− 0.836	0.434 (0.248–0.759)	3.42e−03
SYT5_high–C5AR1_low	− 0.668	0.513 (0.312–0.843)	8.47e−03	− 0.325	0.722 (0.397–1.313)	2.86e−01
SYT5_low–C5AR1_high	− 0.223	0.800 (0.453–1.414)	4.43e−01	− 0.437	0.646 (0.349–1.197)	1.65e−01
SYT5_medium–C5AR1_high	− 0.081	0.922 (0.600–1.418)	7.13e−01	− 0.211	0.810 (0.506–1.296)	3.79e−01

^a^Hazard ratio

^b^Confidence interval

### Estimation of the immune cell composition in the NL and OT IDH-WT glioma clusters

**Fig. 9:**
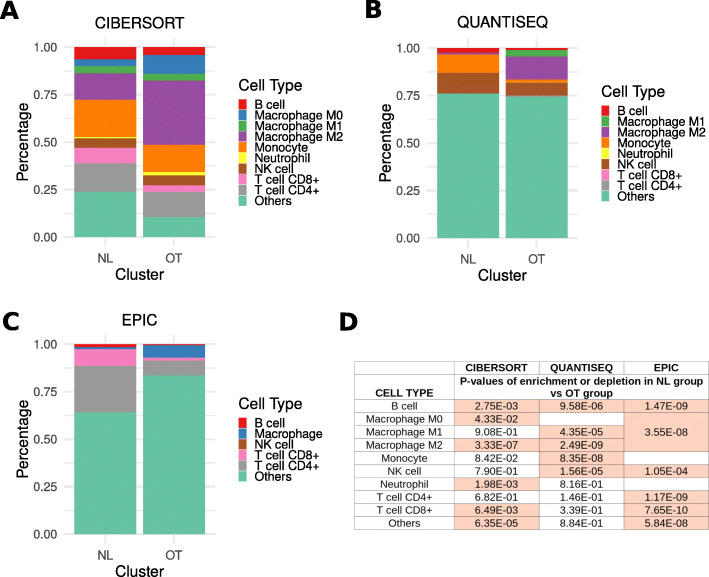
Immune cell type distribution in NL vs OT tumors. Distribution of the immune cell types in NL and OT tumors estimated with Cibersort (**a**), quanTIseq (**b**), and Epic (**c**) software. Statistical analysis was calculated with Mann-Whitney *U* tests (**d**), and the *p* values which are significant (< 0.05) are highlighted in pink

The identification of C5AR1 and MSR1 overexpression in the OT group may suggest differences in the NL vs OT glioma tumor-associated immune microenvironment. We thus explored this hypothesis by inferring the immune cell composition of each glioma sample using Cibersort (Fig. 9a), quanTIseq (Fig. 9b), and Epic (Fig. 9c).

These analyses showed that NL gliomas were associated with a lower count of intratumoral macrophages when compared to the OT gliomas, more specifically with a lower M2 phenotype macrophage number (*p* = 3.33e−07 and *p* = 2.49e−09 for Cibersort and quanTIseq, respectively; Fig. 9d). A higher density of B lymphocytes (*p* = 2.75e−03, *p* = 9.58e−06, and *p* = 1.47e−09 for Cibersort, quanTIseq, and Epic, respectively), NK cells (*p* = 1.56e−05 and *p* = 1.05e−04 for quanTIseq and Epic, respectively), and CD8+ T lymphocytes (*p* = 6.49e−03 and *p* = 7.65e−10 with Cibersort and Epic, respectively) when compared to the OT gliomas was also observed.

## Discussion

Our reanalysis study of TCGA glioma cohort identified 14 IDH-WT gliomas out of 238 possessing a nearly normal transcriptomic profile and associated with fewer significantly deregulated genes than usual IDH-WT gliomas. These NL IDH-WT gliomas were associated with a longer survival interval and a younger age.

They show partial overlap with previously described IDH-WT glioma subcategories using other profiling strategies, such as PA-like astrocytomas [43]⁠ identified from the methylation data and which possess a transcriptomic profile similar to pilocytic astrocytomas of the posterior fossa, uncommon IDH-WT gliomas [17]⁠ identified by cluster of cluster analysis of four data types (mRNA, miRNA, methylation, copy number variation), and molecularly lower-grade gliomas [44]⁠ which are IDH-WT gliomas lacking one of these alterations: EGFR amplification, H3F3A, and pTERT mutations.

The most recent cIMPACT update on IDH-WT infiltrating gliomas recommends upgrading of infiltrating gliomas bearing EGFR amplification and/or 7 gain/10 loss and/or TERT mutation as glioblastoma equivalents. Our reanalysis of TCGA transcriptomic dataset suggests the existence of a minority of infiltrating gliomas bearing these alterations and yet surviving longer than expected. These tumors do not typically bear alterations found in pediatric gliomas (MYB, FGFR1, BRAF V600E-mutant) either (see Additional file 7: Table S2). This suggests that transcriptomic profiling could be used as a complementary method in diagnosing and predicting the outcome for IDH-WT gliomas with unclear histological grading.

The KNN machine learning model supplemented with specific gene filtration steps identified 2-gene expression signatures that detect NL IDH-WT gliomas associated with a significantly longer survival with good performance. The first gene signature was composed of SLC32A1 and MSR1 genes and the second of C5AR1 and SYT5 genes.

The SLC32A1 gene codes for a gamma-aminobutyric acid (GABA) and glycine vesicular transporter. GABA is the main synaptic inhibitory neurotransmitter in the mature human central nervous system. It has been shown that endogenous GABA has an inhibitory effect on glioma cell proliferation and migration during brain development [46]⁠.

The SYT5 gene codes for synaptotagmin 5 which is a membrane protein with a role in neurosecretory vesicle recruitment and exocytosis following cell depolarization and calcium entry. Its involvement in gliomagenesis remains unclear, but it does play a central role in brain neurotransmission [47, 48]⁠⁠. Interestingly, recent work shows that gliomas “hijack” glutaminergic signaling to promote their growth and progression through membrane electric potential firings. Aberrant GABAergic signaling may serve as a deleterious defect that reduces this electrical activity in glioma cells and counteracts their aggressive biology [49, 50]⁠.

The C5AR1 gene, coding for the G protein-coupled receptor for complement component 5a, plays an important role in the innate immunity regulation and tolerance and may be linked to immune checkpoints, as they relate to lung cancers. C5a complement proteins have also been shown to regulate cancer cell migration, proliferation, and angiogenesis [51, 52]⁠. PD-1 (programmed death-1) and its ligand PD-L1 are known drug targets in lung adenocarcinoma and melanoma [53, 54]⁠. The proposed mechanism involves the reactivation of cytotoxic T cells following neoplastic antigen recognition. A synergistic effect of PD-1/PD-L1 and C5A pathways has been proposed and might represent a novel target in potentiating immunity in different cancers, including gliomas [55]⁠.

The class A macrophage scavenger receptor (MSR1 or CD204 gene) is expressed by tumor-associated M2 macrophages that induce tumor progression and angiogenesis by suppressing immunity in the tumor microenvironment [56]⁠. The discovery of CD204 underexpression in normal-like IDH-WT is in line with findings from other studies, supporting the notion that CD204 expression is correlated with worse survival in cancer, including IDH-WT gliomas [57–59]⁠.

The C5A G protein-coupled receptor 1 is known to be expressed on immune cells (such as T cells and macrophage [60])⁠ and on non-myeloid cells (reactive astrocyte, microglia [61, 62]⁠). Activation of this membrane receptor by the C5A ligand has been linked to an increase of the M2 phenotype macrophage population in tumor [63]⁠. This macrophage subpopulation, which also expresses MSR1 receptors, is well known for its pro-tumoral properties in infiltrating gliomas [64]⁠. C5A1 receptor activation is also associated with decreased NK and CD4+/CD8+ T cell responses, known for their pro-inflammatory and anti-tumoral effects [65, 66]⁠. This results in an immunotolerant tumor microenvironment that favors infiltrating glioma progression.

Overall, we envisage that the lower expression of C5AR1 in NL gliomas, by impacting negatively on MSR1-expressing M2 phenotype macrophages and positively on NK and CD4+/CD8+ T cells, favors anti-inflammatory and anti-tumoral cell signaling cascades. This would result in diminished aggressivity.

These findings may suggest the presence of an immunological advantage in atypical NL IDH-WT gliomas which would impact negatively on neoplastic progression. Altered GABA and calcium-signaling events may also participate. Furthermore, the correlation of SLC32A1/MSR1 and C5AR1/SYT5 gene signatures with survival could potentially translate into clinical practice as a personalized protein or nucleic acid-based predictive tool which complements the actual work-up (EGFR amplification, TERT promoter mutation, chr 7 gain/chr 10 loss, and MGMT promoter methylation) in predicting aggressive behavior for IDH-WT infiltrating gliomas and would ensure better patient care.

Recent findings showed the formation of chemical synapses between GBM tumor cells and non-neoplastic cells in the surrounding tumor microenvironment that provide a direct mean of regulating cell invasiveness [67]⁠, following the release of the amino acid transmitter glutamate, which happens to be a GABA precursor. It will be interesting to further decipher the signaling elements involved in these novel cancer-controlling processes, beyond classical ion fluxes and channel openings, and to integrate the metabolic nature of these amino acids in the overall picture.

## Conclusion

In summary, this reanalysis of TCGA IDH-WT glioma expression dataset identified a subgroup of IDH-WT gliomas with an almost normal transcriptomic profile and a longer survival. These NL IDH-WT gliomas, which tend to occur in younger patients, bear fewer genomic mutations and alterations such as EGFR amplifications, chromosome 7/10 alterations, and TERT mutations although some would still qualify as diffuse astrocytic glioma with molecular features of glioblastoma (WHO grade IV). A machine learning-based approach identified C5AR1/SYT5 and MSR1/SLC32A1 signatures which were able to discriminate NL IDH-WT gliomas with high sensitivity and specificity in various glioma expression datasets. In addition to offering some patients a better outlook, these novel transcriptional patterns could offer clues to the development of emerging therapies focused on targeting immune checkpoints and amino acid signaling in gliomas.

## Supplementary information

**Additional file 1: Figure S1.** Pipeline used for the transcriptomic profiling of the different IDH-WT glioma types. Description of the different gene filtration data processing steps (log2 standardization) used for the unsupervised clustering.

**Additional file 2: Figure S2.** Gene signature identification pipeline from the TCGA transcriptomic dataset. A Random Forest was used for the feature extraction (using MDG or Mean Decrease Gini values) and a K Nearest Neighbors algorithm was performed for each 1-gene/2-gene/3gene combinations.

**Additional file 3: Figure S3.** Gene signature validation pipeline. (A) Data extraction and processing methodology for CGGA datasets. IDH-WT gliomas were extracted and then standardized with a log2. (B) Identification of the clusters of interest from the CGGA datasets. A KNN model was trained on the expression TCGA dataset associated with a gene signature.

**Additional file 4: Table S1.** Stromal, immune and tumor purity score estimation using ESTIMATE.

**Additional file 5: Figure S4.** ROC curves associated with the gene signature classifications. ROC curves associated with SLC32A1/MSR1 gene signature generated from the training (A) and testing set (B); ROC curves associated with C5AR1/SYT5 gene signature generated from the training (C) and testing set (D).

**Additional file 6: Figure S5.** Martingale residuals of the null Cox model of the SLC32A1, SYT5, MSR1 and C5AR1 genes.

**Additional file 7: Table S2.** Genomic alterations of the NL IDH-WT tumors.

## Data Availability

The data used in the study are available in TCGA and CGGA public databases (https://portal.gdc.cancer.gov; http://www.cgga.org.cn/). The datasets supporting the conclusions of this article are included within the article (and its additional files).

## Abbreviations

CGGA: Chinese Glioma Genome Atlas
cIMPACT-NOW: Consortium to Inform Molecular and Practical Approaches to CNS Tumor Taxonomy
CNS: Central nervous system
GBM: Glioblastoma multiforme
IDH-WT: Isocitrate deshydrogenase-wild type
IGAP: Ivy Glioblastoma Atlas Project
KNN: *K*-nearest neighbor
KPS: Karnofsky performance status
LGG: Low-grade glioma
NL: Normal-like
OT: Other-type
ROC: Receiver operating characteristic
TCGA: The Cancer Genome Atlas
WHO: World Health Organization
