# Supplementary figures and images for: A machine learning analysis of a “normal-like” IDH-WT diffuse glioma transcriptomic subgroup associated with prolonged survival reveals novel immune and neurotransmitter-related actionable targets
Source: BMC Med. 2020 Oct 16;18:280. doi: 10.1186/s12916-020-01748-x (PMC7565364; doi:10.1186/s12916-020-01748-x)

## ADDITIONAL FILE 1 : FIGURE S1

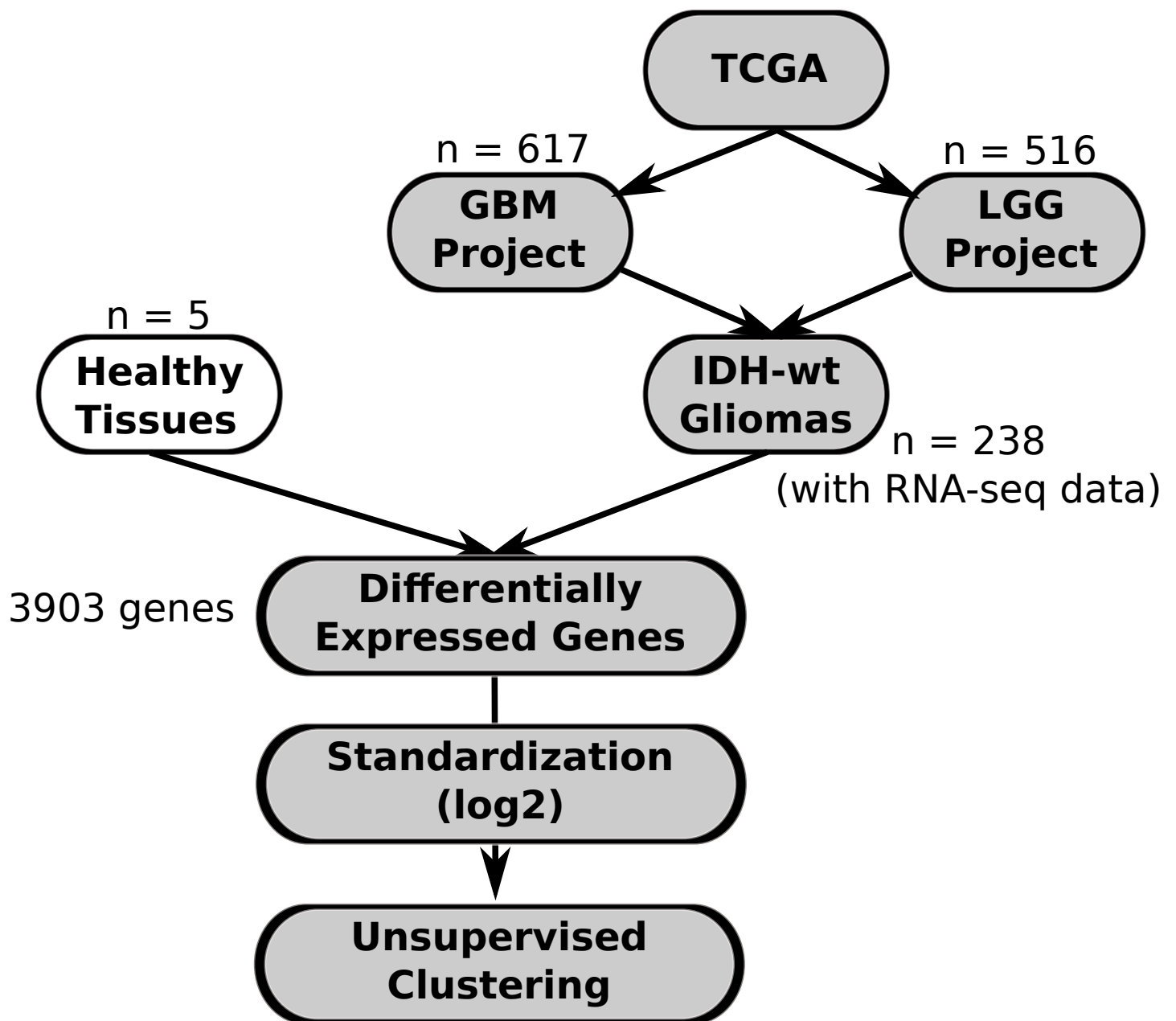

Supplement: Supplementary file 1 — Additional file 1: Figure S1. Pipeline used for the transcriptomic profiling of the different IDH-WT glioma types. Description of the different gene filtration data processing steps (log2 standardization) used for the unsupervised clustering. [file 12916_2020_1748_MOESM1_ESM.pdf]

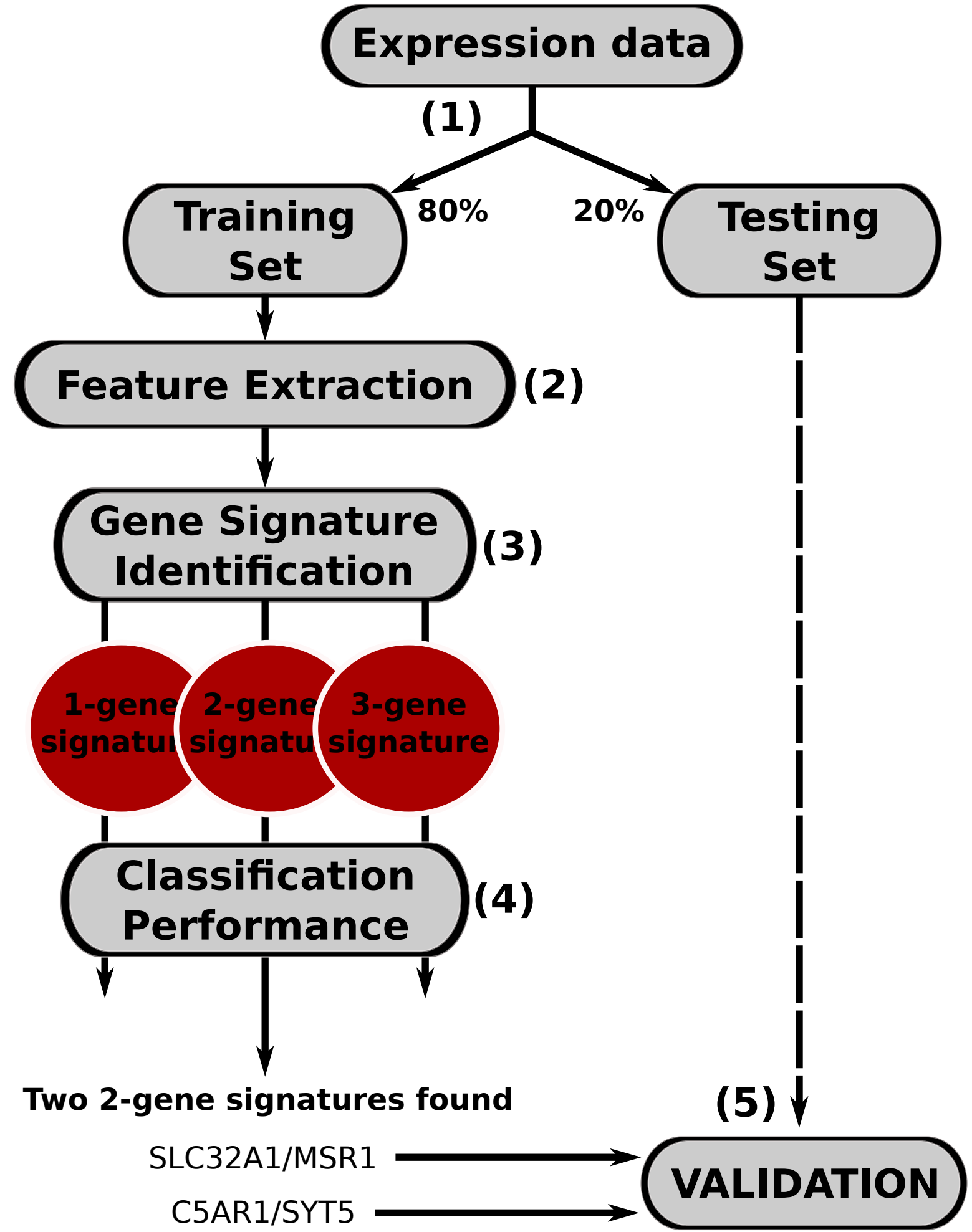

Supplement: Supplementary file 2 — Additional file 2: Figure S2. Gene signature identification pipeline from the TCGA transcriptomic dataset. A Random Forest was used for the feature extraction (using MDG or Mean Decrease Gini values) and a K Nearest Neighbors algorithm was performed for each 1-gene/2-gene/3gene combinations. [file 12916_2020_1748_MOESM2_ESM.pdf]

## ADDITIONAL FILE 3 : FIGURE S3

**A**

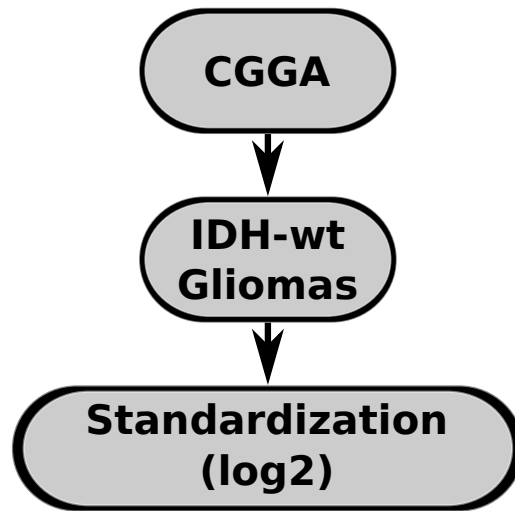

**B**

**K Nearest Neighbors Model**

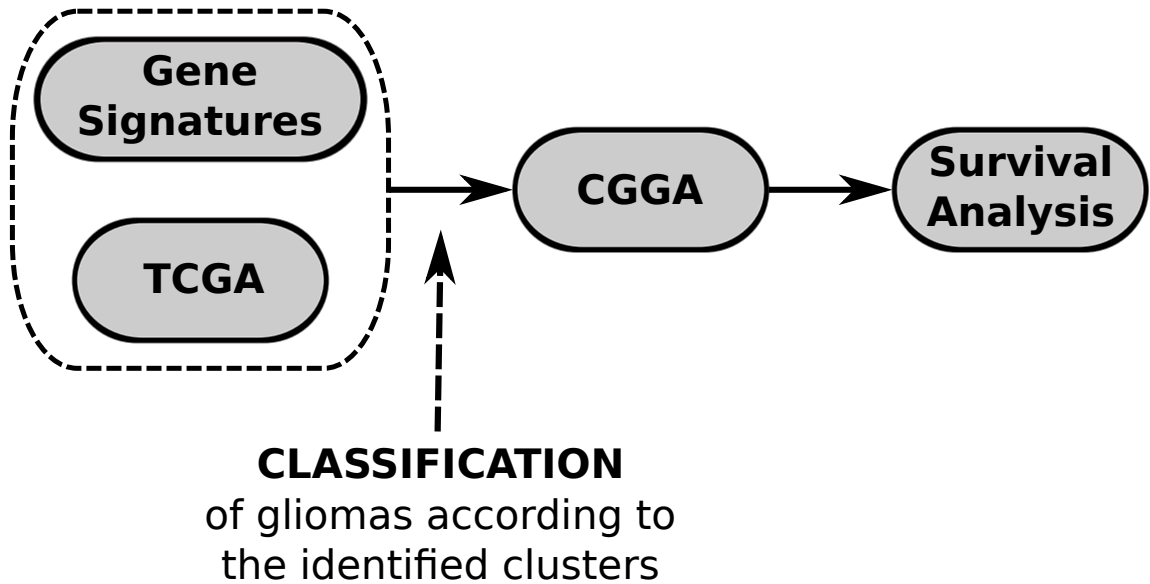

Supplement: Supplementary file 3 — Additional file 3: Figure S3. Gene signature validation pipeline. (A) Data extraction and processing methodology for CGGA datasets. IDH-WT gliomas were extracted and then standardized with a log2. (B) Identification of the clusters of interest from the CGGA datasets. A KNN model was trained on the expression TCGA dataset associated with a gene signature. [file 12916_2020_1748_MOESM3_ESM.pdf]

ADDITIONAL FILE 5 : FIGURE S4

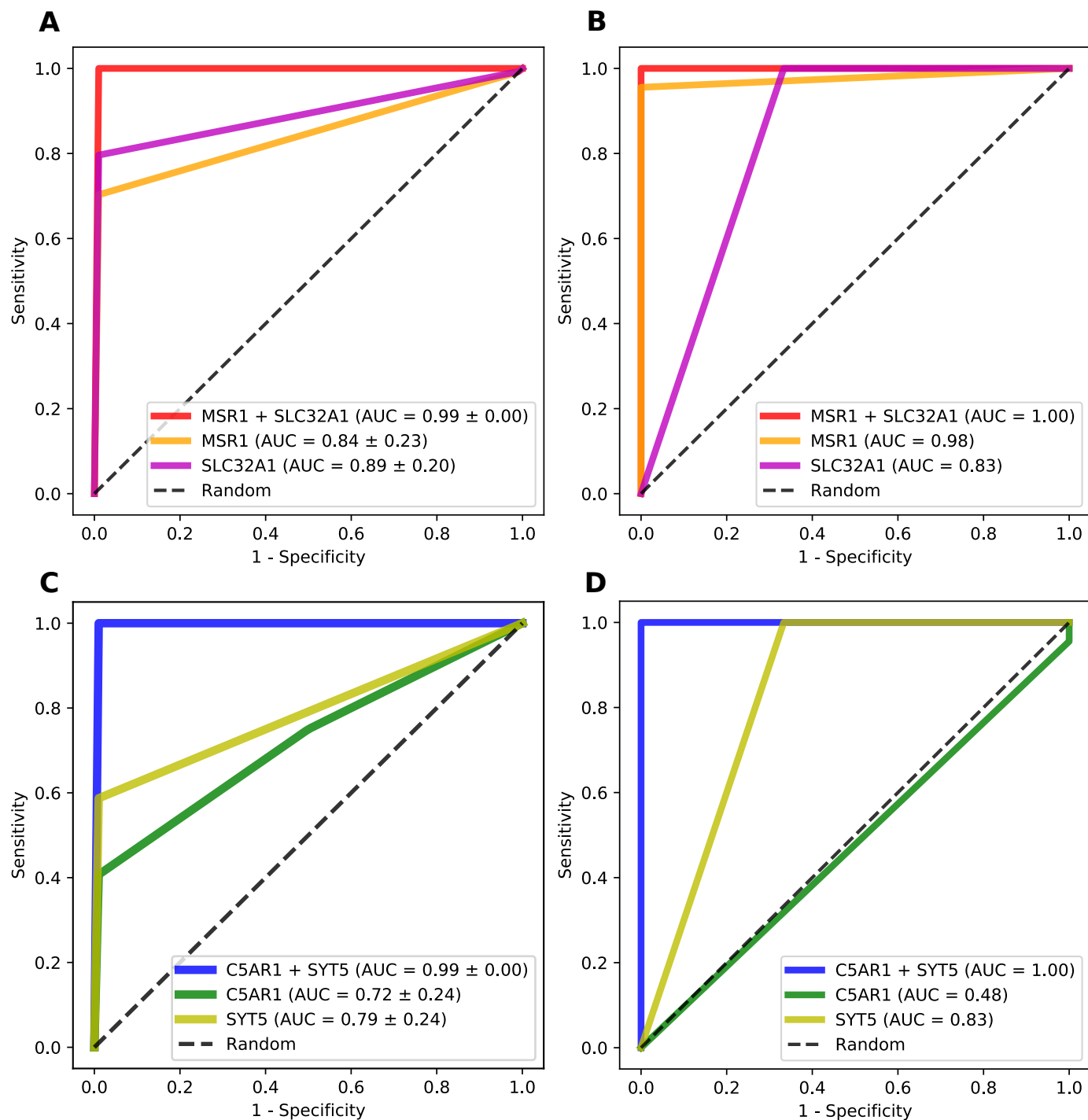

Supplement: Supplementary file 5 — Additional file 5: Figure S4. ROC curves associated with the gene signature classifications. ROC curves associated with SLC32A1/MSR1 gene signature generated from the training (A) and testing set (B); ROC curves associated with C5AR1/SYT5 gene signature generated from the training (C) and testing set (D). [file 12916_2020_1748_MOESM5_ESM.pdf]

ADDITIONAL FILE 6 : FIGURE S5

Martingale Residuals  
of Null Cox Model

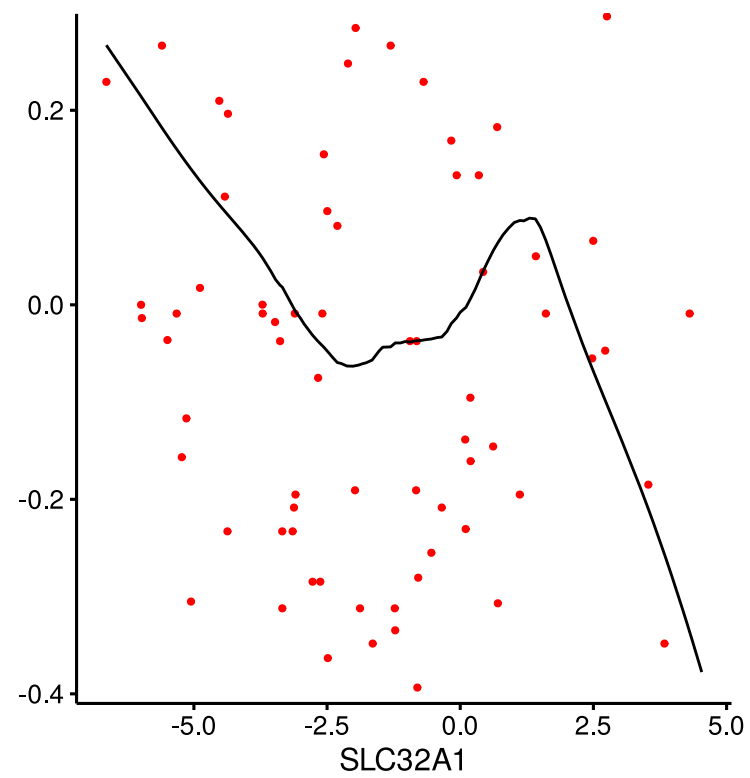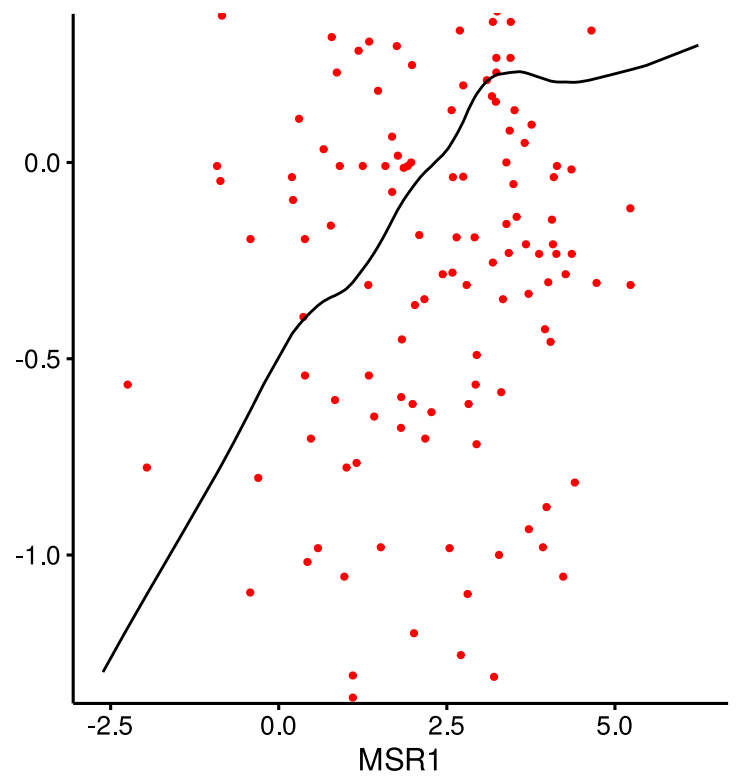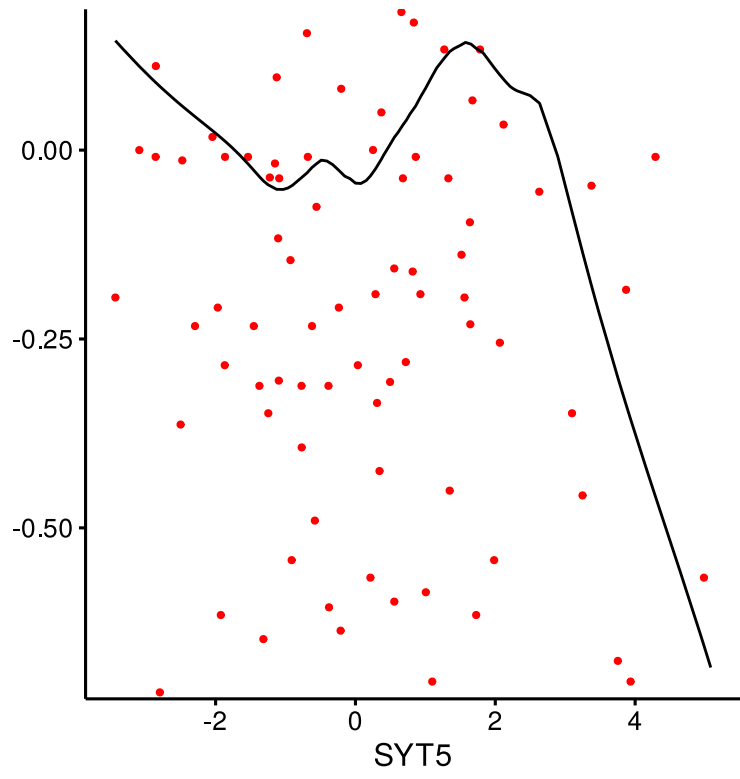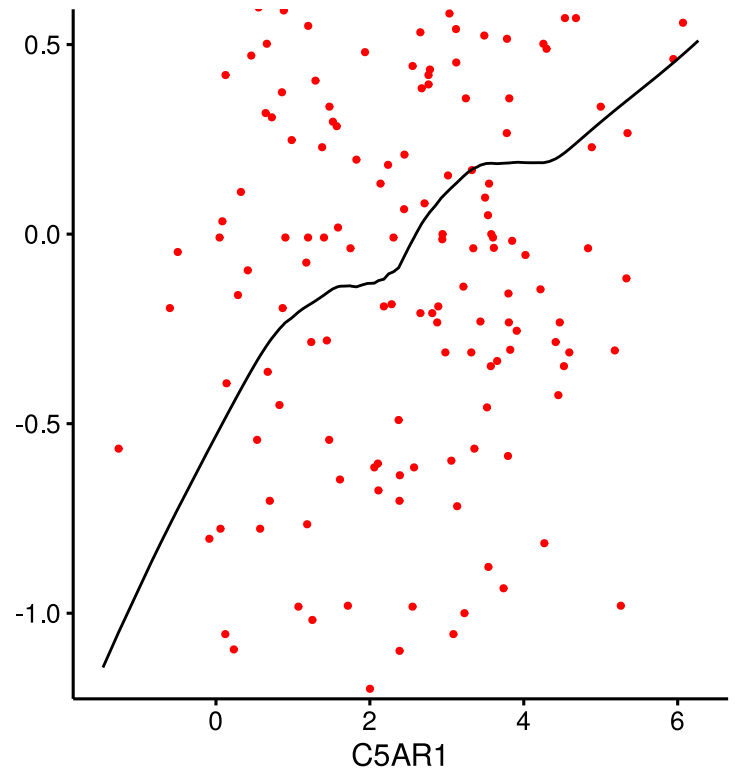

Supplement: Supplementary file 6 — Additional file 6: Figure S5. Martingale residuals of the null Cox model of the SLC32A1, SYT5, MSR1 and C5AR1 genes. [file 12916_2020_1748_MOESM6_ESM.pdf]
